# Telitacicept versus mycophenolate mofetil in IgA nephropathy: a real-world comparative study of efficacy, renal outcomes and safety

**DOI:** 10.1093/ckj/sfaf261

**Published:** 2025-08-12

**Authors:** Yangyang He, Shasha Chen, Kaixiang Liu, Xintong Wu, Min Yu, Wei Wang, Kun Peng, Li Wang, Guisen Li, Xisheng Xie, Wei Qin, Xiang Zhong

**Affiliations:** Department of Nephrology and Institute of Nephrology, Sichuan Provincial People's Hospital, School of Medicine, University of Electronic Science and Technology of China, Chengdu, Sichuan, China; Department of Nephrology and Institute of Nephrology, Sichuan Provincial People's Hospital, School of Medicine, University of Electronic Science and Technology of China, Chengdu, Sichuan, China; Department of Nephrology and Institute of Nephrology, Sichuan Provincial People's Hospital, School of Medicine, University of Electronic Science and Technology of China, Chengdu, Sichuan, China; School of Medicine, University of Electronic Science and Technology of China, Chengdu, Sichuan, China; Department of Nephrology and Institute of Nephrology, Sichuan Provincial People's Hospital, School of Medicine, University of Electronic Science and Technology of China, Chengdu, Sichuan, China; Department of Nephrology and Institute of Nephrology, Sichuan Provincial People's Hospital, School of Medicine, University of Electronic Science and Technology of China, Chengdu, Sichuan, China; Department of Nephrology and Institute of Nephrology, Sichuan Provincial People's Hospital, School of Medicine, University of Electronic Science and Technology of China, Chengdu, Sichuan, China; Department of Nephrology and Institute of Nephrology, Sichuan Provincial People's Hospital, School of Medicine, University of Electronic Science and Technology of China, Chengdu, Sichuan, China; Department of Nephrology and Institute of Nephrology, Sichuan Provincial People's Hospital, School of Medicine, University of Electronic Science and Technology of China, Chengdu, Sichuan, China; Department of Nephrology, Second Clinical College of Nanchong North Sichuan Medical College, Nanchong Central Hospital, Nanchong, Sichuan, China; Division of Nephrology, Department of Medicine, West China Hospital, Sichuan University, Chengdu, Sichuan, China; Department of Nephrology and Institute of Nephrology, Sichuan Provincial People's Hospital, School of Medicine, University of Electronic Science and Technology of China, Chengdu, Sichuan, China

**Keywords:** eGFR, IgA nephropathy, mycophenolate mofetil, proteinuria, telitacicept

## Abstract

**Background:**

This study aimed to evaluate the efficacy and safety of telitacicept versus mycophenolate mofetil (MMF) in high-risk progressive immunoglobulin A nephropathy (IgAN).

**Methods:**

This retrospective, multicentre cohort study included patients with high-risk progressive IgAN who received telitacicept or MMF therapy, both combined with low-dose steroids. Clinical data were collected from treatment initiation to 12 months.

**Results:**

A total of 104 patients were included, with 56 receiving MMF and 48 receiving telitacicept. The average age was 36.9 ± 11.8 years. Baseline characteristics were well balanced between groups, except for serum albumin, uric acid and tubular pathology based on the Oxford classification, which showed significant differences. At 12 months, telitacicept plus low-dose steroids demonstrated superior proteinuria reduction (−62.5% versus −52.9%, *P* = .041) and stabilized renal function (4.1% improvement in estimated glomerular filtration rate versus 5.3% decline with MMF, *P* = .085). Telitacicept plus low-dose steroids achieved higher complete remission rates (33.3% versus 16.1%; *P* = .04) and significantly lower non-response rates (29.2% versus 48.2%, *P* = .048) compared with MMF plus low-dose steroids. Cumulative remission rates (complete + partial) favoured telitacicept at all time points, with the largest difference at 12 months. Notably, telitacicept required substantially lower cumulative glucocorticoid doses (*P* < .001) and exhibited a superior safety profile, with significantly fewer adverse events (22.9% versus 42.9%, *P* = .032) and no serious complications reported. Multivariable analysis indicated telitacicept was associated with a higher likelihood of achieving 12-month complete remission [adjusted hazard ratio 6 (95% confidence interval 1.41–25.62).

**Conclusions:**

Telitacicept may offer better efficacy compared with MMF for proteinuria reduction in high-risk IgAN patients, while reducing combined glucocorticoid requirements and demonstrating a more favourable safety profile. These advantages position it as a promising therapeutic option, warranting further randomized validation.

KEY LEARNING POINTS **What was known:**

- Before this study, mycophenolate mofetil (MMF) was China's primary but variably effective progressive immunoglobulin A nephropathy (IgAN) treatment, with side effects.
- Telitacicept shows promise in reducing proteinuria (phase 2 randomized controlled trial), but lacks real-world comparison data with MMF on efficacy, renal outcomes and safety, creating clinical uncertainty.

**This study adds:**

- Telitacicept may offer better efficacy compared with MMF for proteinuria reduction in high-risk IgAN patients, while reducing combined glucocorticoid requirements and demonstrating a more favourable safety profile.
- This real-world evidence supports considering telitacicept as an alternative to conventional immunosuppressants, particularly for patients prioritizing renal protection and tolerability.

**Potential impact:**

- Telitacicept may emerge as a preferred alternative to MMF for IgAN, offering better renal protection and fewer infections, potentially shifting the treatment strategy for progressive IgAN patients.

## INTRODUCTION

Immunoglobulin A nephropathy (IgAN) is characterized by dominant or co-dominant IgA deposition in the mesangium and is recognized as the most common primary glomerulonephritis (GN) worldwide [1]. The prevalence of IgAN is closely related to geographic distribution, with the highest incidence in the Asia-Pacific region and the lowest in African countries [2]. In China, IgAN accounts for 45.3% of primary GN cases [3]. Most patients with IgAN progress to end-stage kidney disease, making it a leading cause of kidney failure [4]. It is well accepted that all patients with primary IgAN should receive optimized supportive therapy, including lifestyle modifications, blood pressure (BP) management, maximal tolerated renin–angiotensin–aldosterone system (RAAS) blockade and minimization of cardiovascular risk [5]. However, despite maximal supportive care, some patients are still at high risk of progression and treatment is still a challenge. Mycophenolate mofetil (MMF), as a glucocorticoid-sparing agent, is widely used for the treatment of IgAN in China [6–9]. However, some studies have found a higher rate of infection in patients treated with MMF [10–13]. Therefore, there is an urgent need to explore other drugs that are both safe and effective in slowing the progression of IgAN.

The pathogenesis of IgAN is well known as the multihit hypothesis, and the first hit is the production of galactose-deficient IgA1 (Gd-IgA1). B cell activating factor (BAFF/BLys) and its cognate, a proliferation-inducing ligand (APRIL), play a crucial role in both T cell–dependent and independent class switching of B cells to produce Gd-IgA1 [14]. In recent years, therapeutic strategies targeting BAFF/APRIL blockade have shown promising potential in the treatment of IgAN and are considered a potentially effective approach for managing this disease [15]. Telitacicept, as a BAFF/APRIL inhibitor, is a fusion protein that combines a recombinant transmembrane activator and calcium-modulating cyclophilin ligand interactor (TACI) receptor fused to a fragment crystallizable (Fc) domain of human immunoglobulin G (IgG) [16]. Telitacicept is currently approved in China for the treatment of B cell–mediated immune disorders, specifically active systemic lupus erythematosus (SLE) [17] and rheumatoid arthritis (RA) [18]. The first clinic trial of telitacicept in treating IgAN (NCT04291781) was conducted in China. Telitacicept significantly reduced proteinuria and maintained a stable estimated glomerular filtration rate (eGFR) as well as having a good safety in IgAN patients [15]. Later, the US Food and Drug Administration (FDA) approved telitacicept for phase 3 clinical trials in the USA for the treatment of IgAN. In the real world, patients with high-risk progressive IgAN are often advised to participate in clinical trials or consider immunosuppressive therapies, including novel agents such as telitacicept. Therefore, we initiated a retrospective, real-world study that aims to compare the efficacy and safety of telitacicept versus MMF in the treatment of patients with progressive IgAN in China, evaluating which treatment is superior during the 12-month follow-up period.

## MATERIALS AND METHODS

### Study population

This was a retrospective, multicentre cohort study. A total of 605 patients diagnosed with IgAN by renal biopsy at Sichuan Provincial People's Hospital, West China Hospital and Nanchong Central Hospital between November 2021 and November 2023 were screened. A total of 104 patients with IgAN followed for ≥12 months who were receiving MMF (*n* = 56) or telitacicept (*n* = 48) combined with low-dose steroids, with proteinuria >0.75 g/day after receiving standard supportive treatment for ≥3 months and eGFR >30 ml/min/1.73 m^2^ [calculated by the Chronic Kidney Disease Epidemiology Collaboration (CKD-EPI) creatinine equation] [19] were included in the study. Patients with autoimmune diseases, systemic infections or malignancies or who were pregnant, lactating, minors or had received other immunosuppressive agents were excluded from this study. Permission for this retrospective study was given by the Ethics Committee of Sichuan Provincial People's Hospital (trial no. 101, 2024).

### Follow-up and outcome measures

Clinical and follow-up data were collected from historical records. General patient information, including sex, age, body mass index (BMI) and clinical manifestations such as systolic BP, diastolic BP were collected. The changes in 24-h urine proteinuria (proteinuria was measured via 24-h urine collection throughout the study), serum creatinine (SCr), eGFR, uric acid (UA), serum albumin (Alb) and adverse events at 3, 6, 9 and 12 months after treatment initiation were recorded during outpatient follow-up.

The primary endpoints were reduction of proteinuria and eGFR and secondary endpoints were complete clinical remission (CR), partial remission (PR) and non-response (NR). The rate of CR and PR was compared between the two groups at 3, 6, 9 and 12 months. CR was defined as patients with 24-h proteinuria ≤0.3 g, Alb >35 g/l and stable renal function (a decrease in eGFR ≤30%). PR was defined as patients who experienced a >50% reduction from baseline and <1 g/24 h in 24-h proteinuria while maintaining stable renal function but did not achieve CR [20]. NR was defined as not meeting the criteria for CR or PR. eGFR was calculated using the CKD-EPI 2009 equation for adults [19].

### Statistical analysis

Statistical analyses were performed using SPSS 22.0 (IBM, Armonk, NY, USA) and R 3.3.2 (R Foundation for Statistical Computing, Vienna, Austria). Continuous variables were presented as mean ± standard deviation (SD; normal distribution) or median with interquartile range (IQR; skewed distribution), while categorical data were expressed as percentages. Multiple imputation was applied for covariates with missing values (<10%). The variables were compared using the chi-squared test or Fisher's exact test (categorical variables), the Student's *t*-test (continuous variables) or the Kruskal–Wallis (skewed distribution) test. Multivariable Cox regression models were constructed to estimate adjusted hazard ratios (HRs) for 12-month CR + PR. Three models were utilized: model 1 was adjusted for age, sex, BMI, hypertension, proteinuria, eGFR, haemoglobin and UA; model 2 included all variables from model 1 plus M, E, S, T, C scores; and model 3 included all variables from model 2 along with the cumulative dose of steroids. Additionally, we performed subgroup analyses using stratified Cox proportional hazards models based on baseline characteristics, including age (<35 years, ≥35 years), sex, BMI (<25 kg/m^2^, ≥25 kg/m^2^), proteinuria (<1.5 g/day, ≥1.5 g/day), eGFR (<60 ml/min/1.73 m^2^, ≥60 ml/min/1.73 m^2^), M score (M0, M1), S score (S0, S1, S2) and C score (C0, C1, C2), which are presented in a forest plot. Interactions among subgroups were assessed using the likelihood ratio test. Propensity score matching was performed between the MMF and telitacicept groups using a multivariable logistic regression model, with covariates selected to generate propensity scores. A 1:1 nearest neighbour matching algorithm (calliper width 0.01) was applied and standardized mean differences were calculated to assess balance. Baseline covariate differences were evaluated using paired *t*-tests (continuous variables) and chi-squared tests (categorical variables). The propensity scores were incorporated as weights in pairwise algorithmic and standardized mortality ratio weighting models to adjust for baseline confounders, enhancing the validity of the treatment–mortality association. Sensitivity to unmeasured confounding was quantified using E-values. Time-to-event data were employed to evaluate the cumulative probability of the 12-month CR rate and the combined CR and PR rate using Kaplan–Meier curves and the between-group difference was compared using the logrank test. Two-sided *P*-values <.05 were considered statistically significant.

## RESULTS

### Baseline characteristics

**Figure 1:**
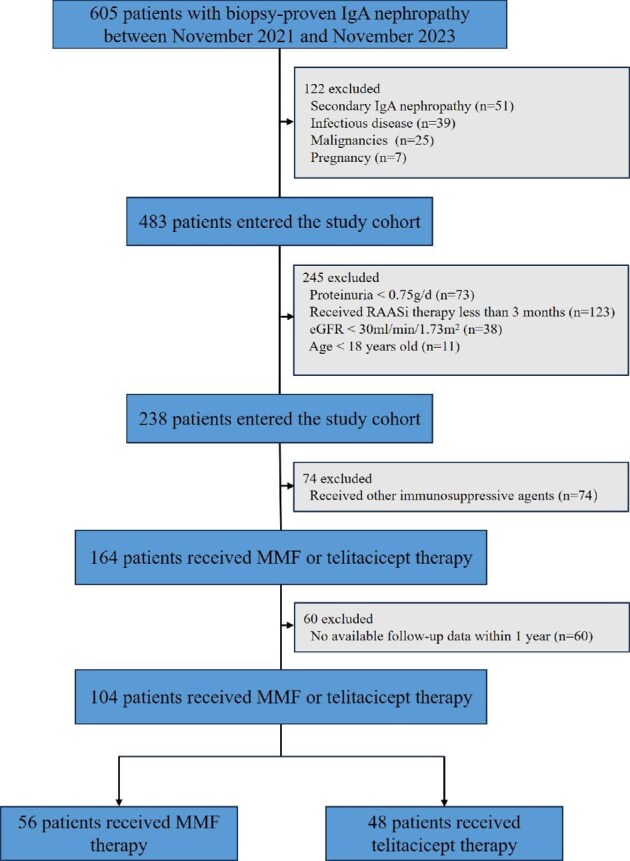
Flow chart of the trial. RAASi: renin–angiotensin–aldosterone system inhibitor.

In this study, 605 patients with IgAN confirmed by renal biopsy were screened and 104 patients were ultimately included in the analysis: 56 received MMF and 48 received telitacicept (Fig. 1). The mean age of the patients was 36.9 ± 11.8 years, with no significant difference between the treatment groups (*P* = .625). Overall, the baseline characteristics were well balanced between the groups. However, significant differences were observed in serum albumin (MMF: 36.8 ± 4.6 g/l; telitacicept: 40.5 ± 3.6 g/l, *P* < .001), uric acid (MMF: 430.1 ± 90.9 µmol/l; telitacicept: 369.4 ± 115.4 µmol/l, *P* = .003) and more advanced tubular pathology based on the Oxford classification in the telitacicept group (*P* = .022) (Table 1, Fig. 1).

**Table 1:** Baseline characteristics of patients.

Variables	Total (*N* = 104)	MMF (*n* = 56)	Telitacicept (*n* = 48)	*P*-value
Age (years), mean ± SD	36.9 ± 11.8	37.4 ± 12.7	36.3 ± 10.8	.625
Sex, *n* (%)				.144
Female	57 (54.8)	27 (48.2)	30 (62.5)	
Male	47 (45.2)	29 (51.8)	18 (37.5)	
Systolic BP (mmHg), mean ± SD	122.5 ± 10.3	122.4 ± 10.9	122.6 ± 9.8	.912
Diastolic BP (mmHg), mean ± SD	76.7 ± 6.8	76.0 ± 8.0	77.4 ± 5.2	.282
BMI (kg/m^2^), mean ± SD	23.6 ± 3.6	23.5 ± 3.6	23.6 ± 3.6	.842
BUN (mmol/l), mean ± SD	7.0 ± 2.7	7.1 ± 2.6	6.9 ± 2.8	.813
Serum albumin (g/l), mean ± SD	38.5 ± 4.6	36.8 ± 4.6	40.5 ± 3.6	<.001
UA (mmol/l), mean ± SD	402.1 ± 106.8	430.1 ± 90.9	369.4 ± 115.4	.003
Haemoglobin (g/l), mean ± SD	131.0 ± 20.4	129.1 ± 24.5	133.1 ± 14.3	.331
Proteinuria (g/day), median (IQR)	1.7 (1.2, 2.7)	1.7 (1.1, 2.8)	1.6 (1.3, 2.8)	.958
eGFR (ml/min/1.73 m^2^), median (IQR)	64.5 (47.6–93.3)	68.4 (47.1–90.6)	71.4 (46.8–96.4)	.632
SCr (μmol/l), mean ± SD	115.6 ± 51.0	120.9 ± 53.6	109.5 ± 47.7	.261
Concomitant medications, *n* (%)				
RAASi	94 (90.4)	52 (92.9)	42(87.5)	.507
SGLT2i	22 (21.2)	10 (17.9)	12 (25.0)	.472
Oxford histological score, *n* (%)				
M				.157
0	66 (63.5)	39 (69.6)	27 (56.2)	
1	38 (36.5)	17 (30.4)	21 (43.8)	
E				.766
0	73 (70.2)	40 (71.4)	33 (68.8)	
1	31 (29.8)	16 (28.6)	15 (31.2)	
S				.579
0	42 (40.4)	24 (42.9)	18 (37.5)	
1	62 (59.6)	32 (57.1)	30 (62.5)	
T				.022
0	85 (81.7)	51 (91.1)	34 (70.8)	
1	14 (13.5)	3 (5.4)	11 (22.9)	
2	5 ( 4.8)	2 (3.6)	3 (6.2)	
C				.87
0	47 (45.6)	24 (42.9)	23 (48.9)	
1	53 (51.5)	30 (53.6)	24 (50.0)	
2	3 ( 2.9)	2 (3.6)	1 (2.1)	

BUN: blood urea nitrogen; RAASi: renin–angiotensin–aldosterone system inhibitor; SGLT2i: sodium–glucose co-transporter 2 inhibitor; M: mesangial hypercellularity (M0, <50% of glomeruli show mesangial hypercellularity; M1, >50% of glomeruli show mesangial hypercellularity); E: endocapillary hypercellularity (E0, no endocapillary hypercellularity; E1, any glomeruli show endocapillary hypercellularity); S: segmental glomerulosclerosis (S0, absent; S1, present in any glomeruli); T: tubular atrophy/interstitial fibrosis (T0, 0–25% of cortical area; T1, 26–50% of cortical area; T2, >50% of cortical area); C: crescents (C0, absent; C1, 0–25% of glomeruli; C2, ≥25% of glomeruli).

### Treatment regimen

In this real-world study, the dose of MMF varied from 1.0 to 1.5 g/day (0.5 to 0.75 g every 12 h) during the follow-up. Telitacicept was subcutaneously injected with a dose of 160–240 mg/week. All patients received a low dose (0–0.6 mg/kg/day) of oral prednisone for 1–2 months and were then tapered by 20% every month for the next 2–4 months in both groups. Cumulative prednisone doses over 12 months were 2.94 ± 0.18 g for MMF versus 0.66 ± 0.148 g for telitacicept (*P* < .001). Stable and optimized RAAS inhibitor therapy, including angiotensin-converting enzyme inhibitor (ACEi) or angiotensin II receptor blocker (ARB), and sodium–glucose co-transporter 2 inhibitor (SGLT2i) was also administered in both groups. Trimethoprim/sulfamethoxazole (TMP/SMX) prophylaxis was administered to 32 patients (57.1%) in the MMF group, while no patients (0%) in the telitacicept group received this prophylaxis (*P* < .001). Through the 12-month follow-up, the median treatment duration was 5.0 months (IQR 3.3–7.0) for telitacicept and 12.0 months (IQR 8.0–12.0) for MMF (*P* < .001). In the telitacicept group (*n* = 48), no patients completed the full 12-month course due to protocol-defined success criteria or socio-economic factors: 45.8% (22/48) achieved CR or PR with early termination per protocol, 45.8% (22/48) discontinued due to financial constraints (lack of insurance coverage in China) and 8.3% (4/48) followed legacy phase 2 trial protocols (24-week regimen). In contrast, 57.1% (32/56) of MMF patients completed the 12-month therapy without discontinuation (Supplementary Table S3).

### Treatment outcome

**Figure 2:**
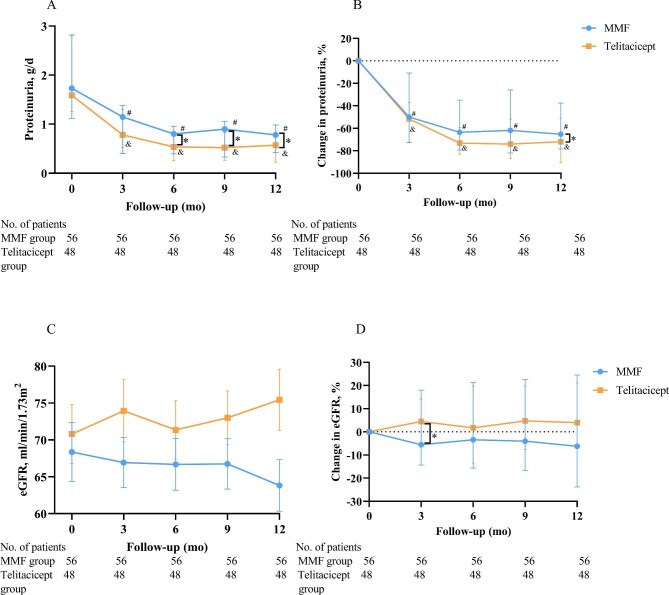
**(A)** Proteinuria and **(B)** the change in proteinuria from baseline in the MMF and telitacicept groups at follow-up. The median and IQR are shown. **(C)** eGFR and **(D)** the change in eGFR from baseline in the MMF and telitacicept groups at follow-up. ^#^*P* < .05 versus baseline in the MMF group; ^&^*P* < .05 versus baseline in the telitacicept group; *P < .05 comparison between groups.

At 6 months after treatment initiation, proteinuria levels in the MMF and telitacicept plus steroids groups decreased significantly from baseline [MMF: 0.8 g/day (IQR 0.4–1.0) versus 1.7 g/day (IQR 1.1–2.8), *P* < .001; telitacicept: 0.5 g/day (IQR 0.3–0.8) versus 1.6 g/day (IQR 1.3–2.8), *P* < .001; Fig. 2A). From 6 to 12 months after treatment initiation, proteinuria levels remained relatively stable in the two groups. There were differences in proteinuria between the two groups, with significant differences observed at 6 months [0.5 g/day (IQR 0.3–0.8) versus 0.8 g/day (IQR 0.4–1.0), *P* = .031], 9 months [0.5 g/day (IQR 0.3–0.8) versus 0.9 g/day (IQR 0.3–1.1), *P* = .033] and 12 months [0.6 g/day (IQR 0.2–0.7) versus 0.8 g/day (IQR 0.4–1.0, *P* = .015] in the telitacicept and MMF groups. Within 9 months of treatment, the changes in proteinuria were similar between the two groups. At the end of the follow-up, the change in proteinuria in the telitacicept group was reduced to −62.5% (IQR −84.6 to −46.3), a greater reduction than in the MMF group [−52.9% (IQR −78.5 to −37.6), *P* = .041; Fig. 2B].

In the telitacicept group, a slight improvement in eGFR was observed at 12 months compared with baseline [74.3 ml/min/1.73 m^2^ (IQR 54.3–100.6) versus 71.4 ml/min/1.73 m^2^ (IQR 45.0–98.7), *P* = .149; Fig. 2C]. In contrast, the MMF group demonstrated a faster eGFR decline over 12 months than the telitacicept group [64.8 ml/min/1.73 m^2^ (IQR 47.9–80.4) versus 68.4 ml/min/1.73 m^2^ (IQR 47.1–90.6), *P* = .285; Fig. 2C]. The change in eGFR levels in the telitacicept group showed an improvement of eGFR compared with the MMF group at 12 months after treatment [4.1% (IQR −5.4–21.1) versus −5.3% (IQR −23.8–24.5), *P* = .085; Fig. 2D].

**Figure 3:**
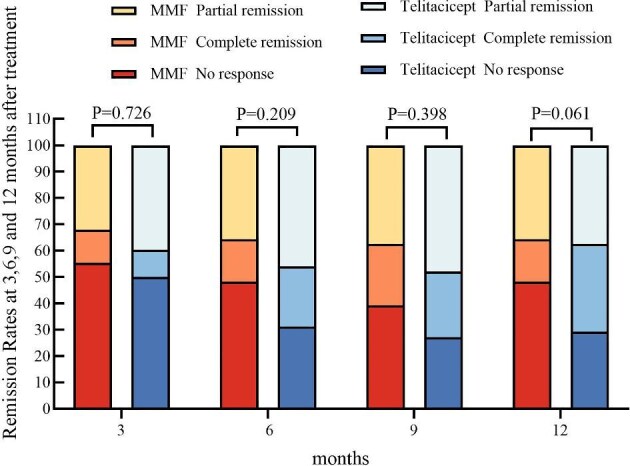
Remission rates at 3, 6, 9 and 12 months after treatment.

Based on the cumulative probability curves and remission rates in Table 2, telitacicept demonstrated a delayed yet clinically significant advantage over MMF in achieving CR, despite initially comparable response rates. While both treatments showed similar CR/PR rates at 3–9 months (*P* > .05), telitacicept exhibited a progressive increase in CR rates (10.4% at 3 months to 33.3% at 12 months) versus stagnation with MMF (12.5% to 16.1%), culminating in a significant superiority at 12 months (*P* = .04). This aligns with the widening separation in the CR curve over time. The steeper attrition in telitacicept's CR + PR curve reflects conversion of partial responders to CR (PR decreased from 39.6% to 37.5% while CR tripled), whereas MMF maintained stable but lower CR rates. Consequently, telitacicept's non-response rate plummeted to 29.2% versus MMF's 48.2% by 12 months, underscoring its enhanced long-term efficacy despite higher early patient dropout in combined response tracking. This pattern suggests telitacicept's biologic mechanism enables sustained remission induction through progressive PR-to-CR conversion (Table 2, Fig. 3).

**Table 2:** Remission rates at 3, 6, 9 and 12 months after treatment.

Variables	Total (*N* = 104)	MMF (n = 56)	Telitacicept (*n* = 48)	*P*-value
3 months, *n* (%)				.726
NR	55 (52.9)	31 (55.4)	24 (50)	
CR	12 (11.5)	7 (12.5)	5 (10.4)	
PR	37 (35.6)	18 (32.1)	19 (39.6)	
6 months, n (%)				.209
NR	42 (40.4)	27 (48.2)	15 (31.2)	
CR	20 (19.2)	9 (16.1)	11 (22.9)	
PR	42 (40.4)	20 (35.7)	22 (45.8)	
9 months, *n* (%)				.398
NR	35 (33.7)	22 (39.3)	13 (27.1)	
CR	25 (24.0)	13 (23.2)	12 (25)	
PR	44 (42.3)	21 (37.5)	23 (47.9)	
12 months, *n* (%)				.061
NR	41 (39.4)	27 (48.2)	14 (29.2)	
CR	25 (24.0)	9 (16.1)	16 (33.3)	
PR	38 (36.5)	20 (35.7)	18 (37.5)	

**Figure 4:**
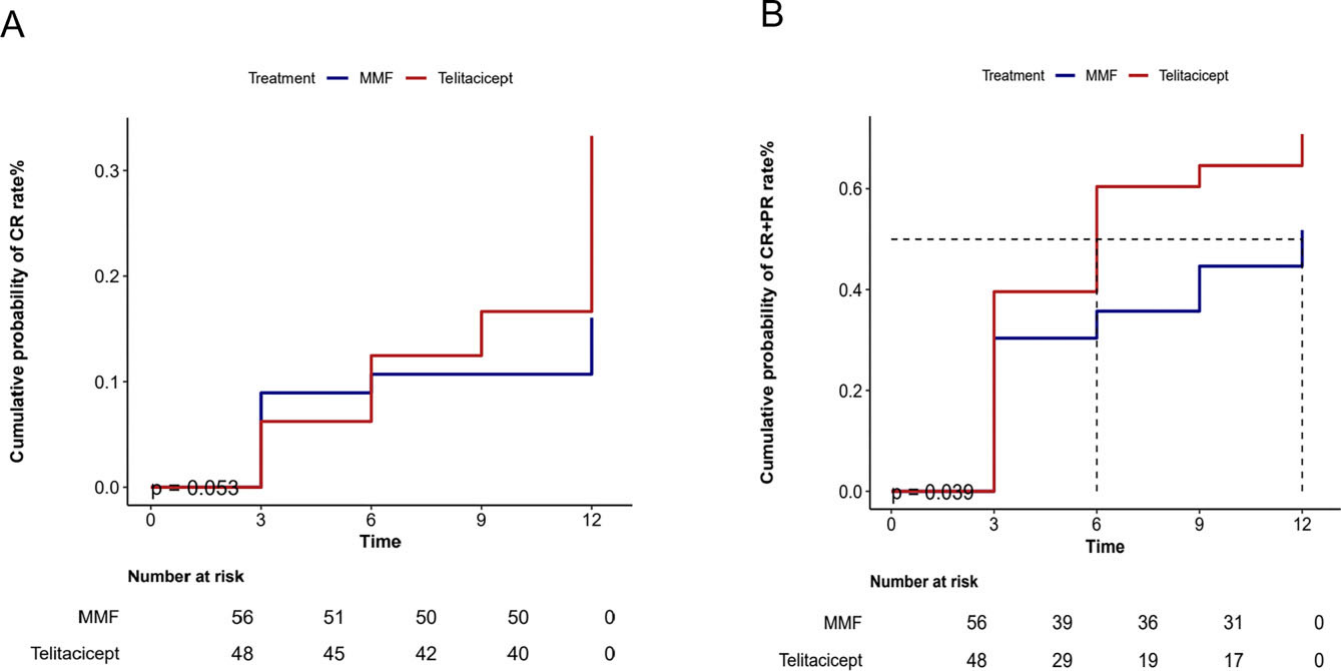
Cumulative probability of 12-month **(A)** CR and **(B)** CR + PR) using Kaplan–Meier analysis.

Fig. 4 illustrates the comparative efficacy of telitacicept versus MMF over time. The CR curve (panel A) demonstrates progressive separation favouring telitacicept after 6 months, aligning with Table 2’s significant improvement in 12-month CR rates (33.3% versus MMF's 16.1%, *P* = .04). Conversely, the CR + PR curve (panel B) exhibits steeper early attrition for telitacicept, reflecting its higher conversion rate of PR to CR over time (PR decreased from 39.6% at 3 months to 37.5% at 12 months while CR tripled) and explaining the divergence from MMF's stable but lower-efficiency profile (Fig. 4).

Supplementary Table S3 details on-treatment drug exposure timelines, showing progressive telitacicept discontinuation (100% at 3 months versus 0% at 12 months) versus sustained MMF use (98.2% at 3 months versus 57.1% at 12 months). Notably, response rates in telitacicept patients remained stable after discontinuation (6-month CR: 22.9% versus 12-month CR: 33.3%), confirming durable therapeutic effects despite shorter exposure.

### Univariate and multivariable Cox regression analyses for 12-month CR rates

Supplementary Table S1 presents the results of univariate Cox regression analysis examining the association between various factors and 12-month CR rates. Confounding variables were selected based on clinical relevance, existing literature and statistical significance in univariate analyses.

Table 3 displays the results of multivariable Cox proportional hazards models assessing the impact of treatment regimen on 12-month CR, with adjustments for various covariates across different models. In the crude model, telitacicept plus steroids showed a higher HR of 12-month CR rates compared with MMF plus steroids [HR 2.2 (95% CI 0.97–4.98), *P* = .059]. This association was significant after adjusting for age, sex, BMI, hypertension, proteinuria, eGFR, haemoglobin and UA [model 1: HR 3.04 (95% CI 1.2–7.68), *P* = .019] and further adjusting for Oxford classification scores (M, E, S, T, C) [model 2: HR 3.07 (95% CI 1.14–8.31), *P* = .027]. The fully adjusted model, including all variables from model 2 plus steroids cumulative dose, also demonstrated telitacicept was associated with a higher likelihood of achieving 12-month CR [adjusted HR 6 (95% CI 1.41–25.62)] (Table 3, Supplementary Table S1).

**Table 3:** Multivariable Cox proportional hazards model analysis of treatment regimen and 12-month CR.

	Crude model		Model 1		Model 2		Model 3	
Variable	HR (95% CI)	*P*-value	HR (95% CI)	*P*-value	HR (95% CI)	*P*-value	HR (95% CI)	*P*-value
MMF	1 (Ref)		1 (Ref)		1 (Ref)		1 (Ref)	
Telitacicept	2.2 (0.97–4.98)	.059	3.04 (1.2–7.68)	.019	3.07 (1.14–8.31)	.027	6 (1.41–25.62)	.016

Crude model: not adjusted;

model 1: adjusted for age, sex, BMI, hypertension, proteinuria, eGFR, haemoglobin and UA;

model 2: adjusted for model 1 plus M, E, S, T, C scores;

model 3: adjusted for model 2 plus cumulative steroids dose.

### Propensity score matching and additional analyses for 12-month CR

To account for confounding, we employed propensity score–based methods. Propensity scores were estimated using logistic regression, incorporating age, sex, BMI, hypertension, proteinuria, eGFR, haemoglobin, UA and Oxford scores. For matching, a 1:1 nearest-neighbour algorithm with a calliper of 0.1 was applied. Weighting methods included inverse probability of treatment weighting (IPTW), proportional allocation weighting and overlap weighting, each designed to balance covariates or address positivity violations. The analysis shows progressively higher HRs with more advanced adjustment methods. The crude model had borderline significance (HR 2.26, *P* = .051), while propensity score–adjusted (HR 9.92, *P* = .002) and IPTW models (HR 13.98, *P* < .001) showed strong effects. However, overlap weighting (HR 4.31, *P* = .016) had wide confidence intervals. These variations suggest treatment effect estimates are highly method dependent, with IPTW showing the greatest precision (Table 4, Supplementary Fig. S1).

**Table 4:** Summary of treatment effect across different adjustment methods.

Models	HR (95% CI)	*P*-value
Unmatched, crude	2.26 (1–5.11)	.051
Multivariable, adjusted	6 (1.41–25.62)	.016
Propensity score, adjusted	9.92 (2.32–42.36)	.002
Propensity score, matched	10.75 (1.22–94.49)	.032
Weighted, IPTW	13.98 (6.14–31.83)	<.001
Weighted, PA	4.07 (0.72–23.12)	.028
Weighted, OW	4.31 (0.56–33.31)	.016

Adjusted for age, sex, BMI, hypertension, proteinuria, eGFR, haemoglobin, UA, cumulative steroids dose and M, E, S, T, C scores.

PA: proportional allocation weighting; OW: overlap weighting.

### Subgroup analysis for 12-month CR

**Figure 5:**
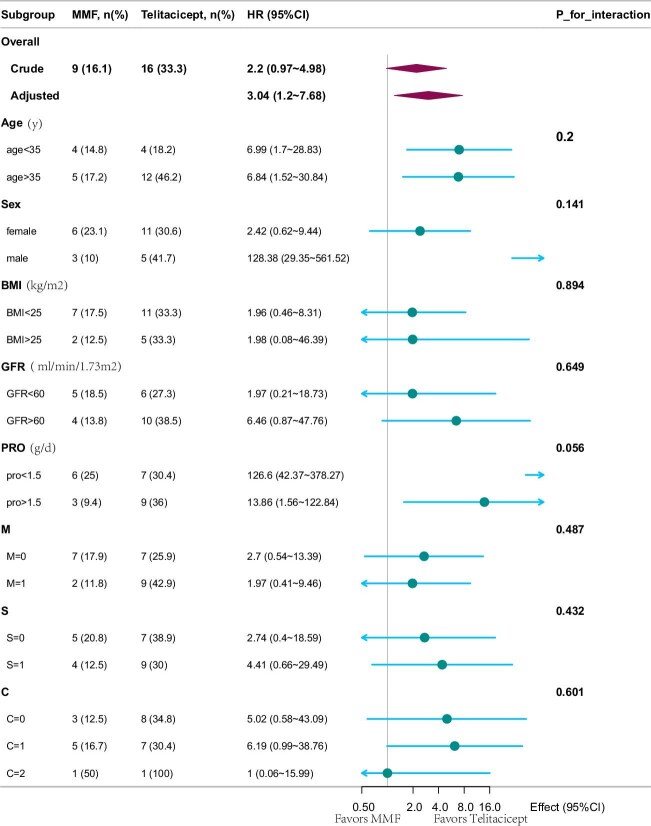
Subgroup analyses of the association between treatment regimen and 12-month CR. Adjusted for age, sex, BMI, proteinuria, eGFR, hypertension, haemoglobin, UA and M, E, S, T, C scores.

Subgroup analyses were conducted based on the fully adjusted model (model 3) from our primary Cox regression. The benefit of telitacicept was consistent across all subgroups (all HRs ≥1 favour telitacicept). Notably, patients with baseline proteinuria ≥1.5 g/day had the strongest effect [HR 13.86 (95% CI 1.56–122.84), *P*-interaction = .056). Although an extreme HR was observed in males (HR 128.38), it was likely due to sparse data rather than biological reasons. In terms of histology, the HR for mesangial hypercellularity (M1) was 1.97, segmental sclerosis (S1) was 4.41 and crescents (C1) was 6.19. Importantly, there were no significant interactions (all *P*-interaction > .05), indicating that the treatment effects of telitacicept were consistent regardless of age, sex, renal function or histology (Fig. 5, Supplementary Table S2).

### Safety and adverse events

MMF plus steroids was associated with a higher overall frequency of adverse events compared with telitacicept (42.9% versus 22.9%, *P* = .032). Specifically, MMF was linked to a greater incidence of fatigue, alopecia and giddiness, which were not observed in the telitacicept group. Conversely, injection site pain was more common among telitacicept-treated patients, likely attributable to its subcutaneous administration route. Notably, no serious adverse events were reported in either group. Overall, telitacicept plus steroids demonstrated a more favourable safety profile, with fewer adverse events reported (Table 5).

**Table 5:** Summary of adverse events.

Events	MMF (*n* = 56)	Telitacicept (*n* = 48)
SAEs, *n* (%)	0	0
Adverse events, *n* (%)		
Fatigue	5 (8.7)	0 (0)
Alopecia	7(12.3)	0 (0)
Nausea	1(1.7)	0 (0)
Diarrhoea	1 (1.7)	1 (2.1)
Cough	4 (7.1)	0 (0)
Expectoration	2 (3.5)	0 (0)
Insomnia	2 (3.5)	0 (0)
Giddy	7 (12.3)	0 (0)
Muscle pain	2 (3.6)	0 (0)
Thrombopenia	1 (1.7)	0 (0)
Rash	4 (7.0)	0 (0)
Itching	2 (3.5)	0 (0)
Urodynia	1 (1.6)	0 (0)
Injection site pain	0 (0)	10 (20.8)

Adjusted for age, sex, BMI, hypertension, proteinuria, eGFR, haemoglobin, UA and M, E, S, T, C scores.

## DISCUSSION

This multicentre, retrospective study describes, for the first time, the real-world clinical experience of IgAN patients treated with telitacicept versus MMF plus steroids over a 12-month follow up. In the present study we demonstrated that telitacicept plus steroids significantly reduces proteinuria with mild adverse reactions in Chinese patients with IgAN during a 12-month follow-up period. Furthermore, telitacicept plus steroids consistently achieved higher remission rates (both complete and partial) and lower non-response rates at 6, 9 and 12 months, with the most significant differences observed at 12 months. Interestingly, after 12 months of follow-up, although there was no statistically significant difference in the change in eGFR between the two groups, eGFR exhibited a more pronounced improvement and kidney protection in the telitacicept group compared with the MMF group.

Currently, targeting BAFF and APRIL is a potentially useful strategy for treating IgAN. BAFF and APRIL are essential for B cell development and survival. BAFF promotes the survival and development of transitional B cells into mature B cells and supports B cell proliferation, plasma cell survival and class transformation and recombination. APRIL promotes cell survival and class transformation and recombination and closely relates to T cell–independent response [21]. Sibeprenlimab, an APRIL-neutralizing agent, has demonstrated significant proteinuria reduction in phase 2 clinical trials for IgAN treatment [22]. Atacicept is a recombinant fusion protein that selectively binds and neutralizes both BAFF and APRIL. In the ORIGIN phase 2b study (NCT04716231) in patients with IgAN, atacicept improved kidney endpoints with a reduction of proteinuria and stabilization of eGFR while reducing serum Gd-IgA1 [23]. Telitacicept, another dual BAFF/APRIL inhibitor, treats IgAN by suppressing B cells that produce Gd-IgA1 and anti-Gd-IgA1 autoantibodies, thereby slowing immune complex deposition [24]. In the phase 2 clinical trial, telitacicept treatment led to a clinically meaningful reduction in proteinuria in patients with IgAN after 6 months of treatment [15]. In a large multicentre retrospective study from 19 sites in China, Liu *et al.* [25] found telitacicept alone or with steroids therapy significantly and safely reduced proteinuria in 97 patients with IgAN when followed up for 6 months. A real-world report including 21 patients over a 3-month follow-up recently found that telitacicept may be an effective treatment for IgAN patients by reducing proteinuria and preserving eGFR and showed a favourable safety profile [26]. However, the long-term efficiency and safety still need to be confirmed.

Different from these studies, this study followed up for 12 months and provided further evidence of telitacicept versus MMF in treatment of IgAN. Through a longer follow-up period, we found that the proteinuria levels in the telitacicept group showed a rapid response at 6 months and continued to decrease from a baseline of 1.6 g/day to 0.6 g/day at 12 months and an improvement in eGFR was observed at 12 months in the telitacicept group. More importantly, telitacicept plus steroids treatment demonstrated a consistent association with a higher likelihood of achieving 12-month CR across different adjustment models. Multivariable analysis indicated telitacicept was associated with a higher likelihood of achieving 12-month CR [adjusted HR 6 (95% CI 1.41–25.62)].

Mycophenolic acid (MPA) is the active component in MMF and inhibits the proliferation of B and T cells by selectively inhibiting inosine monophosphate dehydrogenase, a key enzyme in the purine synthesis pathway. These cells play key roles in the immune response in IgAN. B cells produce abnormal IgA1 antibodies, while T cells are involved in regulating the inflammatory response. MMF can reduce the production of deposited IgA antibodies by inhibiting the activity of these cells, thereby reducing immune-mediated damage in the kidney [27, 28]. Several studies from China have reported that MMF alone or combined with corticosteroids is beneficial in patients with progressive IgAN [9, 29–31]. The comparative efficacy of immunosuppressive agents and telitacicept in the treatment of IgAN remains a topic of ongoing research, with inconsistent conclusions drawn from different studies. A case–control retrospective study from China found telitacicept achieved similar clinical efficacy as conventional immunosuppressive therapy for 24 weeks in reducing proteinuria and increasing eGFR, with fewer adverse effects observed in the telitacicept group [20]. In the recent 3-month follow-up study by Qin *et al.* [26], telitacicept reduced the median proteinuria by 0.72 g/day (54.6% from baseline) while maintaining stable eGFR levels, whereas the immunosuppressive therapy group achieved a greater proteinuria reduction of 1.12 g/day (62.5% from baseline) but showed a concurrent decrease in eGFR. Importantly, telitacicept demonstrated a favourable safety profile with no serious adverse events reported, contrasting with the renal function concerns associated with conventional immunosuppressive regimens [26]. Our results demonstrated that both BAFF/APRIL inhibition with telitacicept and suppression of B and T cell proliferation with MMF were effective in reducing proteinuria in IgAN. However, compared with MMF at 12 months, telitacicept demonstrated statistically greater proteinuria reduction (*P* < .05), significantly higher rates of CR/PR and more pronounced kidney-protective effects. Telitacicept's lower ‘no response’ rate at 12 months (29.2% versus MMF’s 48.2%) supports its long-term advantage despite higher attrition in the CR + PR curve. Future research should focus on identifying patient subgroups that may benefit most from each treatment and on conducting long-term studies to better understand their respective roles in the management of IgAN.

In terms of safety, both treatment groups exhibited good tolerability overall. However, consistent with previous reports, telitacicept was associated with fewer adverse effects during the 12-month follow-up, suggesting that it may be better tolerated in the long term. This favourable safety profile further supports the potential clinical advantages of telitacicept over MMF in the management of IgAN. Meanwhile, the significant difference in medication duration reflects distinct safety profiles and infection risk management strategies, as evidenced by differential prophylaxis utilization in our cohort (MMF 57.1% versus telitacicept 0%, *P* < .001).

Our study provides valuable insights into the efficacy and safety of telitacicept compared with MMF in treating IgAN. However, several limitations should be noted. First, we did not monitor key immune markers (e.g. Gd-IgA1) that could provide deeper insights into the immunomodulatory effects of telitacicept. Second, the retrospective design introduces potential biases. Third, this study included only Chinese patients and the findings may not extrapolate to other ethnic populations. Notably, IgAN exhibits marked heterogeneity across races. For instance, while MMF has demonstrated efficacy in Asian cohorts [9], it failed to show significant benefits in predominantly Caucasian populations [32, 33]. Future research should include multicentre randomized controlled trials with diverse cohorts, longer follow-up and comprehensive immunological assessments to validate our findings and better understand the long-term impact of telitacicept in IgAN.

## CONCLUSION

In this real-world study, telitacicept may offer preferable efficacy compared with MMF for proteinuria reduction in high-risk IgAN patients, while reducing combined glucocorticoid requirements and demonstrating a more favourable safety profile. These findings support telitacicept as a promising therapeutic strategy for balancing sustained renal protection with reduced treatment-related risks in progressive IgAN. Further randomized trials are needed to confirm these observations.

## Supplementary Material

sfaf261_Supplemental_File

## Data Availability

The data underlying this article will be shared upon reasonable request to the corresponding author.

## References

[1] Wyatt RJ, Julian BA. IgA nephropathy. N Engl J Med 2013;368:2402–14. 10.1056/NEJMra1206793 23782179

[2] Stamellou E, Seikrit C, Tang SCW et al. IgA nephropathy. Nat Rev Dis Primers 2023;9:67. 10.1038/s41572-023-00476-9 38036542

[3] Xie Y, Chen X. Epidemiology, major outcomes, risk factors, prevention and management of chronic kidney disease in China. Am J Nephrol 2008;28:1–7. 10.1159/000108755 17890852

[4] Pitcher D, Braddon F, Hendry B et al. Long-term outcomes in IgA nephropathy. Clin J Am Soc Nephrol 2023;18:727–38. 10.2215/CJN.0000000000000135 37055195 PMC10278810

[5] Rovin BH, Adler SG, Barratt J et al. Executive summary of the KDIGO 2021 guideline for the management of glomerular diseases. Kidney Int 2021;100:753–79. 10.1016/j.kint.2021.05.015 34556300

[6] Hou FF, Xie D, Wang J et al. Effectiveness of mycophenolate mofetil among patients with progressive IgA nephropathy: a randomized clinical trial. JAMA Netw Open 2023;6:e2254054. 10.1001/jamanetworkopen.2022.54054 36745456 PMC12578496

[7] Tang S, Leung JC, Chan LY et al. Mycophenolate mofetil alleviates persistent proteinuria in IgA nephropathy. Kidney Int 2005;68:802–12. 10.1111/j.1523-1755.2005.00460.x 16014059

[8] Tang SC, Tang AW, Wong SS et al. Long-term study of mycophenolate mofetil treatment in IgA nephropathy. Kidney Int 2010;77:543–9. 10.1038/ki.2009.499 20032964

[9] Hou JH, Le WB, Chen N et al. Mycophenolate mofetil combined with prednisone versus full-dose prednisone in IgA nephropathy with active proliferative lesions: a randomized controlled trial. Am J Kidney Dis 2017;69:788–95. 10.1053/j.ajkd.2016.11.027 28215945

[10] Dooley MA, Jayne D, Ginzler EM et al. Mycophenolate versus azathioprine as maintenance therapy for lupus nephritis. N Engl J Med 2011;365:1886–95. 10.1056/NEJMoa1014460 22087680

[11] Werth VP, Joly P, Mimouni D et al. Rituximab versus mycophenolate mofetil in patients with pemphigus vulgaris. N Engl J Med 2021;384:2295–305. 10.1056/NEJMoa2028564 34097368

[12] Maes BD, Oyen R, Claes K et al. Mycophenolate mofetil in IgA nephropathy: results of a 3-year prospective placebo-controlled randomized study. Kidney Int 2004;65:1842–9. 10.1111/j.1523-1755.2004.00588.x 15086925

[13] Frisch G, Lin J, Rosenstock J et al. Mycophenolate mofetil (MMF) vs placebo in patients with moderately advanced IgA nephropathy: a double-blind randomized controlled trial. Nephrol Dial Transplant 2005;20:2139–45. 10.1093/ndt/gfh974 16030050

[14] Filippone EJ, Gulati R, Farber JL. Contemporary review of IgA nephropathy. Front Immunol 2024;15:1436923. 10.3389/fimmu.2024.1436923 39188719 PMC11345586

[15] Lv J, Liu L, Hao C et al. Randomized phase 2 trial of telitacicept in patients with IgA nephropathy with persistent proteinuria. Kidney Int Rep 2023;8:499–506. 10.1016/j.ekir.2022.12.014 36938094 PMC10014376

[16] Dhillon S . Telitacicept: first approval. Drugs 2021;81:1671–5. 10.1007/s40265-021-01591-1 34463932

[17] Wu D, Li J, Xu D et al. Telitacicept in patients with active systemic lupus erythematosus: results of a phase 2b, randomised, double-blind, placebo-controlled trial. Ann Rheum Dis 2024;83:475–87. 38129117 10.1136/ard-2023-224854 PMC10958275

[18] Yao X, Ren Y, Zhao Q et al. Pharmacokinetics analysis based on target-mediated drug distribution for RC18, a novel BLyS/APRIL fusion protein to treat systemic lupus erythematosus and rheumatoid arthritis. Eur J Pharm Sci 2021;159:105704. 10.1016/j.ejps.2021.105704 33440243

[19] Levey AS, Stevens LA, Schmid CH et al. A new equation to estimate glomerular filtration rate. Ann Intern Med 2009;150:604–12. 10.7326/0003-4819-150-9-200905050-00006 19414839 PMC2763564

[20] Wang M, Ma J, Yao L et al. Efficacy and safety of telitacicept, a BLyS/APRIL dual inhibitor, in the treatment of IgA nephropathy: a retrospective case–control study. Clin Kidney J 2024;17:sfae285. 10.1093/ckj/sfae285 39391591 PMC11464987

[21] Hoffman W, Lakkis FG, Chalasani G et al. Antibodies, and more. Clin J Am Soc Nephrol 2016;11:137–54. 10.2215/CJN.09430915 26700440 PMC4702236

[22] Mathur M, Barratt J, Chacko B et al. A phase 2 trial of sibeprenlimab in patients with IgA nephropathy. N Engl J Med 2024;390:20–31. 10.1056/NEJMoa2305635 37916620 PMC7615905

[23] Lafayette R, Barbour S, Israni R et al. A phase 2b, randomized, double-blind, placebo-controlled, clinical trial of atacicept for treatment of IgA nephropathy. Kidney Int 2024;105:1306–15. 10.1016/j.kint.2024.03.012 38552841

[24] Zeng L, Yang K, Wu Y et al. Telitacicept: a novel horizon in targeting autoimmunity and rheumatic diseases. J Autoimmun 2024;148:103291. 10.1016/j.jaut.2024.103291 39146891

[25] Liu L, Liu Y, Li J et al. Efficacy and safety of telitacicept in IgA nephropathy: a retrospective, multicenter study. Nephron 2025;149:1–10. 10.1159/000540326 39250892

[26] Dong L, Yang D, Qin A et al. Efficacy and safety of telitacicept in IgA nephropathy: a real-world study. Ren Fail 2025;47:2449580. 10.1080/0886022X.2025.2449580 39780498 PMC11721934

[27] Bhat R, Tonutti A, Timilsina S et al. Perspectives on mycophenolate mofetil in the management of autoimmunity. Clin Rev Allergy Immunol 2023;65:86–100. 37338709 10.1007/s12016-023-08963-3

[28] Coppo R . Biomarkers and targeted new therapies for IgA nephropathy. Pediatr Nephrol 2017;32:725–31. 10.1007/s00467-016-3390-9 27324471

[29] Liang Y, Zhang J, Liu D et al. Retrospective study of mycophenolate mofetil treatment in IgA nephropathy with proliferative pathological phenotype. Chin Med J (Engl) 2014;127:102–8. 10.3760/cma.j.issn.0366-6999.20132396 24384432

[30] Luo MN, Pan Q, Ye T et al. Efficacy and safety of sequential immunosuppressive treatment for severe IgA nephropathy: a retrospective study. Front Pharmacol 2023;14:1093442. 10.3389/fphar.2023.1093442 36998610 PMC10043386

[31] Hou FF, Xie D, Wang J et al. Effectiveness of mycophenolate mofetil among patients with progressive IgA nephropathy. JAMA Netw Open 2023;6:e2254054. 10.1001/jamanetworkopen.2022.54054 36745456 PMC12578496

[32] Rauen T, Eitner F, Fitzner C et al. Intensive supportive care plus immunosuppression in IgA nephropathy. N Engl J Med 2015;373:2225–36. 10.1056/NEJMoa1415463 26630142

[33] Hogg RJ, Bay RC, Jennette JC et al. Randomized controlled trial of mycophenolate mofetil in children, adolescents, and adults with IgA nephropathy. Am J Kidney Dis 2015;66:783–91. 10.1053/j.ajkd.2015.06.013 26209543

