# Supplementary material for: Telitacicept versus mycophenolate mofetil in IgA nephropathy: a real-world comparative study of efficacy, renal outcomes and safety
Source: Clin Kidney J. 2025 Aug 12;18(9):sfaf261. doi: 10.1093/ckj/sfaf261 (PMC12415519; doi:10.1093/ckj/sfaf261)

**Table S1.** Univariate Cox regression analysis for 12-month complete remission.

| Item | HR (95%CI) | P (Wald's test) |
| --- | --- | --- |
| Treatment: Telitacicept vs MMF | 2.2 (0.97,4.98) | 0.059 |
| Age | 1.02 (0.99,1.05) | 0.208 |
| Sex: female vs male | 0.66 (0.29,1.53) | 0.336 |
| Hypertension | 0.76 (0.1,5.58) | 0.783 |
| BMI(Kg/m^2^) | 0.94 (0.83,1.06) | 0.28 |
| ALB(g/L) | 1.09 (1,1.19) | 0.058 |
| Urine acid(umol/L) | 1.0004 (0.9967,1.0041) | 0.839 |
| HB(g/L) | 0.99 (0.97,1.01) | 0.278 |
| Creatinine(umol/L) | 0.9925 (0.9841,1.001) | 0.083 |
| eGFR(ml/min/1.73m^2^) | 1.004 (0.9908,1.0174) | 0.554 |
| Proteinuria(g/d) | 0.85 (0.6,1.21) | 0.368 |
| Oxford score |  |  |
| M score: 1 vs 0 | 1.41 (0.64,3.11) | 0.39 |
| E score: 1 vs 0 | 1.12 (0.48,2.59) | 0.797 |
| S score: 1 vs 0 | 0.72 (0.33,1.58) | 0.411 |
| T score: ref.=0 |  |  |
| 1 | 1.48 (0.55,3.94) | 0.434 |
| 2 | 0 (0,Inf) | 0.997 |
| C score: ref.=0 |  |  |
| 1 | 0.52 (0.25,1.08) | 0.081 |
| 2 | 3.26 (0.75,14.08) | 0.114 |

**Table S2.** Subgroup analyses for 12-month CR using stratified Cox proportional hazards models based on baseline characteristics

| Subgroup | MMF, n(%) | Telitacicept, n(%) | HR (95%CI) | P_for_interaction |
| --- | --- | --- | --- | --- |
| Overall |  |  |  |  |
| Crude | 9 (16.1) | 16 (33.3) | 2.2 (0.97~4.98) |  |
| Adjusted |  |  | 6 (1.41~25.62) |  |
| Age(y) |  |  |  | 0.2 |
| age<35 | 4 (14.8) | 4 (18.2) | 6.99 (1.7~28.83) |  |
| age≥35 | 5 (17.2) | 12 (46.2) | 6.84 (1.52~30.84) |  |
| Sex |  |  |  | 0.141 |
| female | 6 (23.1) | 11 (30.6) | 2.42 (0.62~9.44) |  |
| male | 3 (10) | 5 (41.7) | 128.38 (29.35~561.52) |  |
| BMI(Kg/m^2^) |  |  |  | 0.894 |
| BMI<25 | 7 (17.5) | 11 (33.3) | 1.96 (0.46~8.31) |  |
| BMI≥25 | 2 (12.5) | 5 (33.3) | 1.98 (0.08~46.39) |  |
| eGFR(ml/min/1.73m^2^) |  |  |  | 0.649 |
| eGFR<60 | 5 (18.5) | 6 (27.3) | 1.97 (0.21~18.73) |  |
| eGFR≥60 | 4 (13.8) | 10 (38.5) | 6.46 (0.87~47.76) |  |
| Proteinuria(g/d) |  |  |  | 0.056 |
| pro<1.5 | 6 (25) | 7 (30.4) | 126.6 (42.37~378.27) |  |
| pro≥1.5 | 3 (9.4) | 9 (36) | 13.86 (1.56~122.84) |  |
| M |  |  |  | 0.487 |
| M=0 | 7 (17.9) | 7 (25.9) | 2.7 (0.54~13.39) |  |
| M=1 | 2 (11.8) | 9 (42.9) | 1.97 (0.41~9.46) |  |
| S |  |  |  | 0.432 |
| S=0 | 5 (20.8) | 7 (38.9) | 2.74 (0.4~18.59) |  |
| S=1 | 4 (12.5) | 9 (30) | 4.41 (0.66~29.49) |  |
| C |  |  |  | 0.601 |
| C=0 | 3 (12.5) | 8 (34.8) | 5.02 (0.58~43.09) |  |
| C=1 | 5 (16.7) | 7 (30.4) | 6.19 (0.99~38.76) |  |
| C=2 | 1 (50) | 1 (100) | 1 (0.06~15.99) |  |

Notes: adjusted for age, sex, BMI, hypertension, proteinuria, eGFR, hemoglobine, uric acid, M, E, S, T, C score, steroids cumulative dose.

**Table S3**. On-treatment MMF/Telitacicept exposure timeline at 3, 6, 9, 12 months

|  | MMF | Telitacicept | p |
| --- | --- | --- | --- |
| 3 months | 55（98.2%） | 48（100%） | 0.264 |
| 6 months | 50（89.3%） | 22（45.8%） | ＜0.001 |
| 9 months | 41（73.2%） | 4（8.3%） | ＜0.001 |
| 12 months | 32（57.1%） | 1（0%） | ＜0.001 |

**Figure S1.** Weighting propensity methods for 12-month complete remission rate


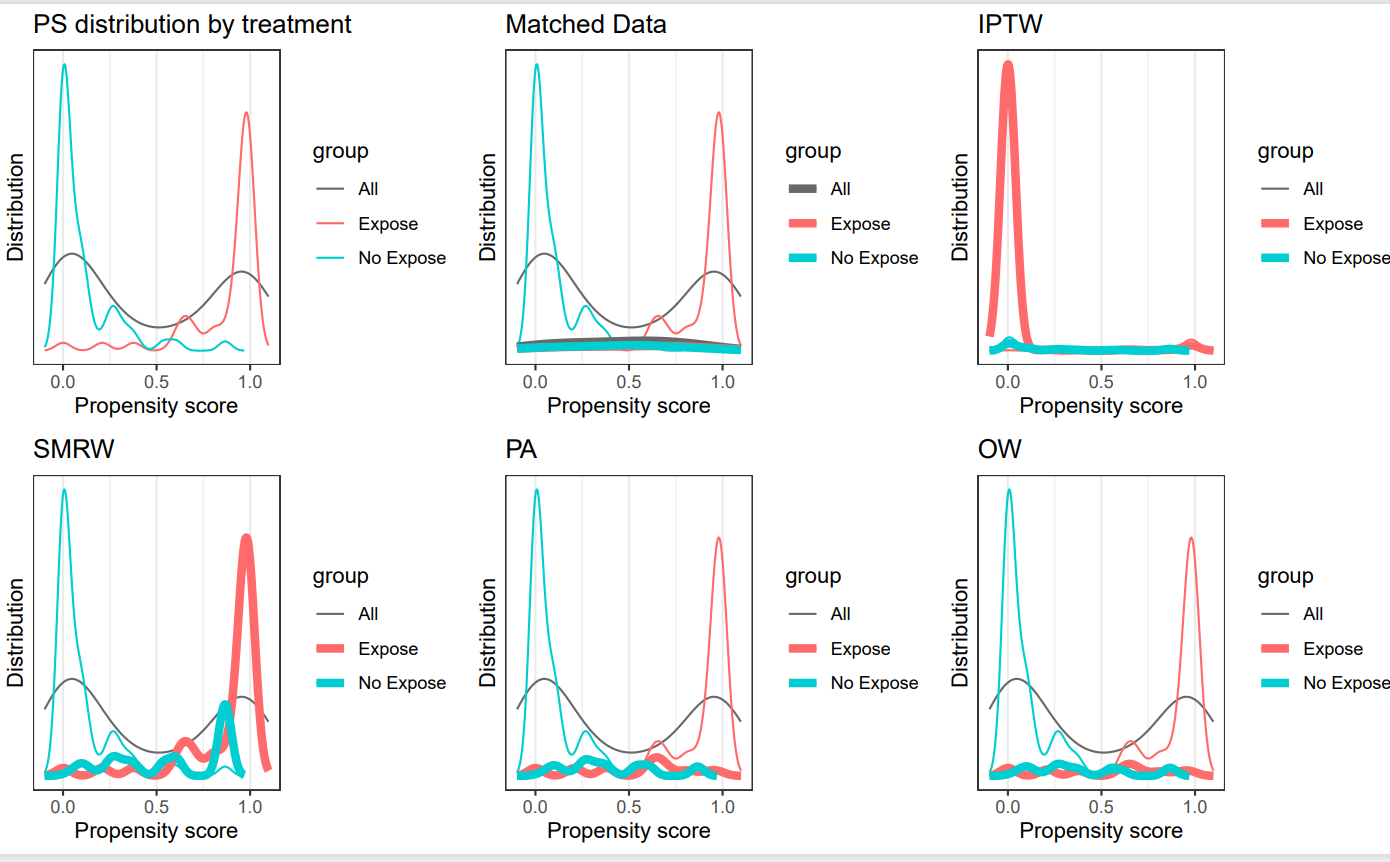

Supplement: sfaf261_Supplemental_File [file sfaf261_supplemental_file.doc]
